# Effect of acupotomy in knee osteoarthritis patients: study protocol for a randomized controlled trial

**DOI:** 10.1186/s13063-021-05247-z

**Published:** 2021-04-20

**Authors:** Danghan Xu, Minghui Lee, Cihui Huang, Jia Wei, Mengxue Zhou, Taotao Yao, Jingjing Lu, Wenjing Zhao, Nuo Xu, Ruina Huang, Jun He, Liang Zheng

**Affiliations:** 1 The First Affiliated Hospital of Guangzhou University of Chinese Medicine, Guangzhou, China; 2 The First Clinical Medical College of Guangzhou University of Chinese Medicine, Guangzhou, China; 3 Kyoto Prefectural University of Medicine, Kyoto, Japan; 4 Shenzhen Pingle Orthopaedic Hospital, Shenzhen, China; 5 The University of Alabama at Birmingham, Birmingham, USA; 6 The Eighth Affiliated Hospital of Sun Yat Sen University, Shenzhen, China

**Keywords:** Acupotomy, Needle-knife, Knee osteoarthritis, Study protocol, Randomized controlled trial

## Abstract

**Background:**

Symptomatic knee osteoarthritis (KOA) is common in China. Pharmacological therapy is not the first recommendation because of its safety issues. Nonpharmacological therapy, such as lifestyle adjustments, weight loss, muscle strengthening, and aerobic exercise programs, is strongly recommended for KOA. However, these approaches may fail due to poor patient compliance. There is a lack of high-quality randomized controlled trials of acupotomy, an effective treatment for KOA. This study was designed to investigate the efficacy of acupotomy in patients with KOA.

**Methods:**

A total of 136 patients will be enrolled at the First Affiliated Hospital of Guangzhou University of Chinese Medicine and assigned to the acupotomy group or sham acupotomy group according to the block randomization scheme. Patients in the acupotomy group will receive 2 sessions of acupotomy for 2 weeks (once a week). Patients in the sham group will receive 2 sessions of sham stimulation for 2 weeks (once a week). All patients will use indomethacin cream externally. The primary outcome will be the Western Ontario and McMaster Universities Osteoarthritis Index (WOMAC), and the secondary outcomes will be the visual analog scale (VAS) score, plantar pressure distribution test result, X-ray examination findings, musculoskeletal ultrasound findings, maximum knee circumference, joint mobility, and quality of life. Measurements will be taken at baseline, 1 week after the end of treatment, and at the 3- and 6-month follow-ups.

**Discussion:**

To the best of our knowledge, this will be the first single-blind, sham-controlled study of acupotomy. The outcome assessors will also be blinded. The aim of this work is to demonstrate the efficacy of acupotomy in treating KOA.

**Trial registration:**

Chinese Clinical Trial Registry ChiCTR2000033047. Registered on 18 May 2020.

**Supplementary Information:**

The online version contains supplementary material available at 10.1186/s13063-021-05247-z.

## Background

As the 11th highest contributor to global disability, knee osteoarthritis (KOA) causes social and familiar burdens [1]. In China, symptomatic KOA is common [2]. Symptomatic KOA causes pain and impaired knee function. A total of 3.9% of KOA patients suffer from severe symptoms, which lead to disability [3]. Pharmacological therapy is not recommended because of its limited efficacy and associated safety issues [4]. Nonpharmacological therapy has been used extensively in treating KOA [5]. For instance, lifestyle adjustments, weight loss, muscle strengthening, and aerobic exercise programs are the recommended methods for first-line treatment [6]. In a systematic review, tai chi was reported to be beneficial in terms of the Western Ontario and McMaster Universities Osteoarthritis Index (WOMAC) (total SMD = -0.41) and in terms of stiffness (total SMD = − 0.20) [7]. However, patient compliance plays an important role in these treatments. At 9 months after stopping exercise, the benefit of exercise in patients with KOA was lost [8]. Thus, we are seeking an effective nonpharmacological therapy with better patient compliance to treat KOA.

Acupotomy is a special type of acutherapy. Unlike standard acupuncture, acupotomy involves a needle-knife with a thicker diameter and flat needle. This needle-knife can enter deep tissues to loosen adhesions. In clinical practice, acupotomy has been mainly used for the treatment of bone diseases and diseases causing chronic soft tissue injury. Acupotomy can alter the balance of mechanical forces around joints, restore the normal state of joint stress, and alleviate inflammation [9, 10]. Therefore, it has a good effect on KOA, frozen shoulder, tennis elbow, and heel pain [11–13]. Acupotomy has been used to treat KOA for more than 30 years [14]. Although previous studies [15–17] have demonstrated the efficacy of acupotomy, it is currently unclear whether acupotomy is an effective treatment for KOA due to a lack of high-quality trials. Therefore, we designed a randomized, single-blind, sham-controlled trial to explore the efficacy of acupotomy in treating KOA.

## Methods

### Study design

**Fig. 1:**
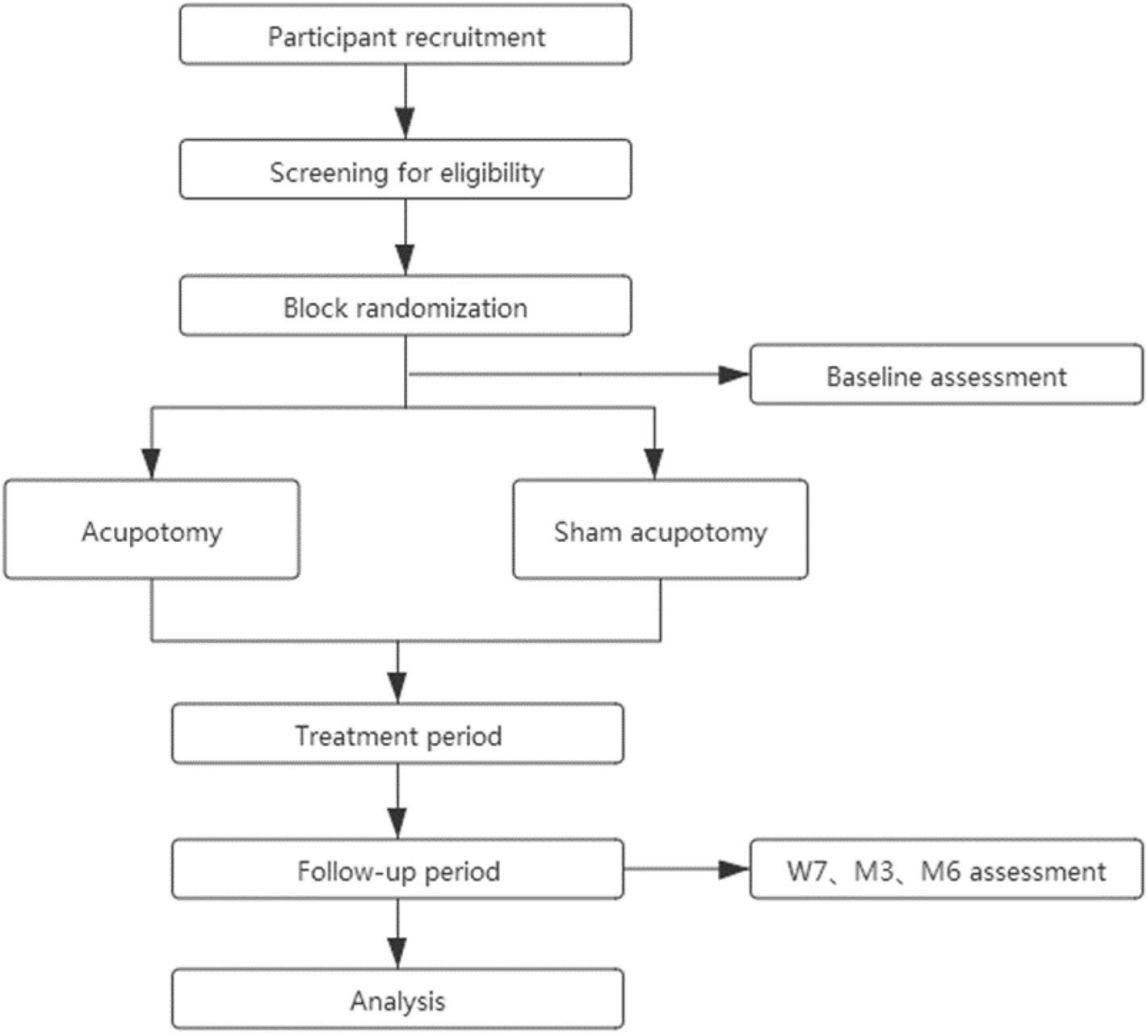
Flowchart of the study design

Based on the Declaration of Helsinki, we designed a parallel, block-randomized, single-blind, sham-controlled trial. The study protocol has been reported in accordance with the Standard Protocol Items: Recommendations for Clinical Interventional Trials (SPIRIT) guidelines [18] (Additional file 1). This study will be performed at the First Affiliated Hospital of Guangzhou University of Chinese Medicine from June 2020 to June 2023 (Fig. 1).

### Participants and recruitment

Participants will be recruited by posting bulletin board advertisements and contacting community doctors. The recruitment information will mainly include eligibility criteria and contact details. A well-trained investigator in the acupuncture department will be responsible for the recruitment of participants. Patients meeting the eligibility criteria will be invited to the clinical research center for further examinations (e.g., X-ray examination, musculoskeletal ultrasound examination, plantar pressure distribution testing and blood analysis), which will be completed within 1 day.

### Inclusion criteria

1. Findings meeting the radiological diagnostic criteria for level I, II, or III KOA based on the Kellgren-Lawrence classification [19];
2. A visual analog scale (VAS) pain score above 4 points in the past month;
3. Age from 40 to 70 years;
4. No use of other related treatment drugs or related treatment methods within 2 weeks;
5. Informed consent.

### Exclusion criteria

1. Pregnancy, lactation, or a plan for pregnancy during the trial;
2. Infectious or serious diseases, such as those affecting the cardiovascular, cerebrovascular, liver, kidney, or hematopoietic systems;
3. Local infection, ulceration, vascular nerve damage, or deep abscess in the knee joint;
4. History of severe knee trauma or surgery or arthroscopy;
5. Other diseases that cause knee pain, such as tumors, knee joint tuberculosis, rheumatoid arthritis, and gouty arthritis;
6. Use of oral corticosteroids or administration of an intra-articular knee injection within 1 month or participation in another clinical trial within 3 months;
7. Allergy to the medical devices involved in this trial;
8. Contraindications to the use of indomethacin plaster, as follows: history of allergy to indomethacin, liver or kidney dysfunction (alanine aminotransferase (ALT) and aspartate aminotransferase (AST) levels more than 2 times the normal value and blood creatinine (Cr) level more than the normal value); asthma, urticaria, or allergic reactions after taking aspirin or other nonsteroidal anti-inflammatory drugs; history of gastrointestinal bleeding or perforation after anti-inflammatory drug use; active gastrointestinal ulcer bleeding, previous recurrent ulcer bleeding; and severe heart failure or history of perioperative coronary artery bypass surgery, etc.;
9. Bleeding tendency, such as long-term use of warfarin, aspirin, and other anticoagulants;
10. History of sedative hypnotic, opioid analgesic, or alcohol abuse;
11. Inability to cooperate with relevant experiments and measurements.

### Intervention

#### Acupotomy group

The patients will be placed in a supine position with the knees flexed 30 ~ 45°. A cushion will be placed beneath the knees. The physicians will select 6 points from a point set, as follows. The point set contains 8 points, including the upper and lower points of the medial collateral ligament of the affected knee joint, the upper and lower points of the lateral collateral ligament, the subpatellar ligament points, the upper point of the patella, the muscle insertion point of the popliteal muscle, and the medial popliteal fossa stimulation points. We will stimulate 6 points for unilateral knee pain and 12 points for bilateral knee pain (Table 1).

**Table 1:** Location of point set

Point name	Location
The upper point of the medial collateral ligament	A lateral tubercle located at the medial condyle of the femur
The lower point of the medial collateral ligament	The posterior part of the liriodendron area located at the medial condyle of the tibia
The upper point of the lateral collateral ligament	A lateral tubercle locating at the lateral condyle of the femur
The lower point of the lateral collateral ligament	The apex of the capitula fibula
The subpatellar ligament points	The lower part of the patellar ligament
The upper point of patella	Directly above the patellar bottom and located in the deep surface of the quadriceps tendon
The insertion of popliteal muscle	Located behind the upper part of the medial tibia
The medial popliteal fossa stimulation points	Located at the medial wall of the popliteal triangle, between the semitendinosus and semimembranosus’ muscles

**Fig. 2:**
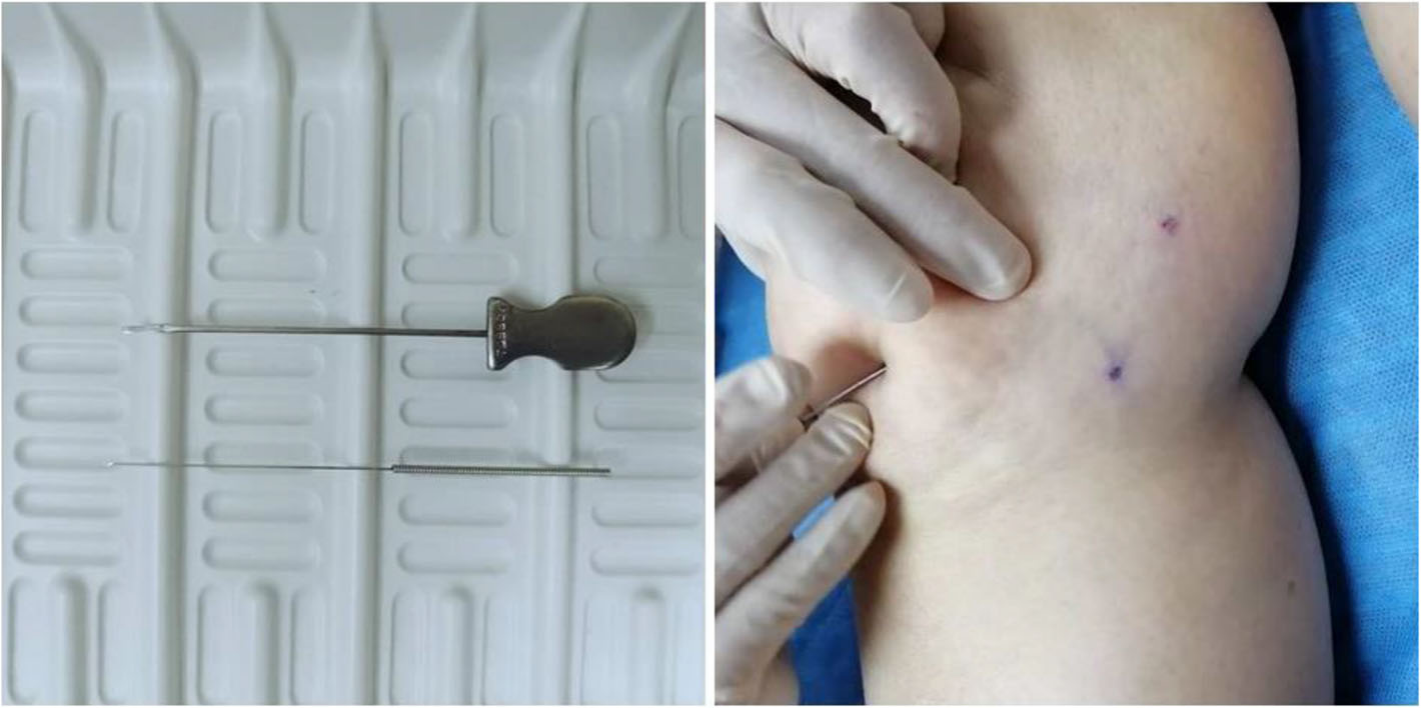
Needle-knife

After standard disinfection, the physicians will operate a Hanzhang type I no. 4 straight needle-knife (Beijing Huaxia Needle Knife Medical Equipment Factory) to perform acupotomy (Fig. 2). Physicians selected will have more than 5 years of experience and will be retrained for 16 h before the study begins.

The incision line will be consistent with the longitudinal axis of the lower limb. The body of the needle-knife will be perpendicular to the skin. The doctor will operate according to the following procedural steps: point fixation, orientation, pressure application, and penetration. When the needle-knife reaches the target depth, the doctor will perform a manual operation. The details of the manual operation are presented in Additional file 2. After the operation, the needle-knife will be pulled out, and local hemostasis will be applied for 3 min. The wound will be disinfected with iodine and dressed with a band aid. Patients will receive 2 acupotomy sessions for 2 weeks (once a week).

#### Sham acupotomy group

Patients assigned to the sham acupotomy group will receive mock acupotomy, which is performed similar to real acupotomy, but without manual stimulation to achieve any real effect. The needle-knife will pierce through the skin only and remain under the skin for 10 s to simulate the manipulation time. Patients will receive 2 sessions of sham acupotomy for 2 weeks (once a week).

Clinicians would give patients indomethacin cream (import drug registration number: H20181060, Nipro Pharma Corporation Saitama Site Plant 2) if the acupotomy treatment is not effective. All patients could receive up to one time of indomethacin cream per day. They can only receive indomethacin cream at most twelve times during the study. The researchers will record the use of indomethacin every day.

### Outcome measurement

We selected both clinical outcomes and surrogate outcomes to assess the efficacy of acupotomy. All outcomes will be measured at baseline and 1 week after the end of treatment. In addition, we will follow-up with patients to evaluate the WOMAC and VAS score at 3 and 6 months after the end of treatment (Table 2). Before the study begins, we will train the related investigators.

**Table 2:** Study schedule. T1-T3: from the first treatment period to the third treatment period. W7: 1 week after the end of treatment. M3: 3 months after the end of treatment. M6: 6 months after the end of treatment

Study schedule						
Period	Screening	Baseline	Treatment	End	Follow-up	
Time	W-1	W0	W1-W6	W7	M3	M6
**Basic**						
Eligibility	√					
Demography	√					
Physical examination	√					
Medical history	√					
Informed consent	√					
**Outcomes**						
WOMAC		√		√	√	√
VAS		√		√	√	√
X-ray examination		√		√		
Musculoskeletal ultrasound imaging		√		√		
Plantar pressure measurement		√		√		
Maximum knee circumference		√		√		
Joint mobility		√		√		
**Trial evaluation**						
Informed consent	√					
Adverse event			√	√		
Safety evaluation			√	√		

### Primary outcome

#### WOMAC

The WOMAC is commonly used to assess KOA symptoms [20]. The scale includes 24 items in three parts, including 5 items related to pain, 2 items related to stiffness, and 17 items related to joint function. The scores are determined using a 10-cm-long visual analog scale beginning with 0 points and ending with 10 points. A score of 0 points indicates no symptoms for that item, and a score of 10 points indicates the most serious degree of symptoms for the item.

### Secondary outcomes

#### VAS score

The VAS score is commonly used to reflect the degree of bodily pain [21]. A 10-cm-long VAS scale beginning with 0 points and ending with 10 points will be used. A score of 0 indicates no pain, and a score of 10 indicate the most severe pain, which is intolerable.

#### Plantar pressure distribution testing

Plantar pressure distribution testing can be used to observe the function of the patients’ knee joints [22]. This test will be performed with the Plantar Pressure Distribution Test System (Belgium RSscan Footscan 1.0 m). A rehabilitation technician will be in charge of the measurement. We will collect balance-related parameters (foot angle and subtalar joint mobility) to assess the stability of the medial and lateral knee joint, with larger values for balance-related parameters indicating poorer stability. In addition, an impulse-related parameter, i.e., the ratio of the heel and forefoot, will be used to analyze knee buffering stability, with larger ratios indicated better stability.

#### X-ray examination

Each patient’s front and lateral knee X-rays will be examined by a physician experienced with imaging. This examination will be performed with the aim of identifying osteophyte formation, joint space narrowing, subchondral sclerosis, cartilage degeneration, osteoporosis, valgus deformity, and varus deformity.

#### Musculoskeletal ultrasound

Ultrasonography has also been proven to be a useful tool in monitoring the efficacy of treatments for KOA [23]. Specialized ultrasound doctors will collect data using a Philip HP Sonos 5500 ultrasound imaging machine. We will focus on the knee joint fluid volume and synovial film thickness. These data will indicate the degree of damage and repair of the knee.

#### Maximum knee circumference

This indicator is used to assess soft tissue lesions around the knee joint [24]. When conducting the assessment, the patients will be asked to lie supine with the knee joints straight. The doctor will use a measuring tape to measure the circumference of the knee joint at the level of the upper and lower poles of the patella, at the middle of the patella, and at other points around the knee joint.

#### Joint mobility

Joint mobility is also used to assess knee function [25]. An arthrometer will be used to measure knee mobility. The patient being tested will be positioned on his/her side with the affected leg being measured facing upward. First, the lateral femoral condyle will be positioned over the center of rotation of the arthrometer. Then, the fixed arm will be positioned along the center of the femur, with the movable arm along the fibula. Last, the patient will extend the knee joint as much as possible and then bend the knee as much as possible, and the range of motion under flexion and extension will be measured by movement of the movable arm.

#### Quality of life (36-Item Short-Form Health Survey (SF-36))

The SF-36 is a concise health questionnaire developed by the Boston Institute of Health to evaluate quality of life. The scale consists of 36 items, including a physical component summary and a mental component summary. The physical health component includes four dimensions: physical function, physical role function, bodily pain, and general health. The mental health component includes four dimensions: vitality, social function, emotional role function, and mental health. Additionally, there will be a section regarding health transitions to evaluate health changes in the past year. The scale of the SF-36 is 0–100, and the score is used to evaluate the quality of life of the subjects, with higher final scores indicating better quality of life [26].

### Sample size

Referring to a previous study [27], we determined that the change in the WOMAC after compared with before treatment in the acupotomy group was 16.34 ± 4.19, and the change in the WOMAC after compared with before treatment in the sham acupotomy group was 14.2 ± 4.19; thus, the effect size was 2.14 [28]. The sample size should provide 80% statistical power at a significance level of 0.05.

We calculated a sample size of 124 with a repeated measurement design using the SAS software package (version 9.4, Tokyo, Japan). Considering a dropout rate of 10% during the research, we decided to enroll 136 participants.

### Randomization

A biostatistician will generate a block randomized number list at a ratio of 1:1 using the Stata 14.0 software package. The block size will not be known until the end of the study for allocation concealment. An independent staff member who will not take part in the performance of this study will seal the random numbers in opaque envelopes. Then, the primary investigator will save these envelopes and open one of them when a participant is enrolled.

### Blinding

This study will follow a single-blind approach. The operators will perform a mock standard acupotomy procedure in the sham acupotomy group. The operators will pierce through the skin only and not stimulate the relevant ligaments and muscles. The needle-knife will stay under the skin for 10 s without any manipulation. At the same time, others involved in this experiment will be blind to the group allocation (data administrators, biostatisticians, programmers, measurement evaluators, etc.) to minimize performance bias.

To protect the rights of the subjects, we will approve free acupotomy for patients in the sham acupotomy group at the end of the trial.

### Date management and monitoring

We will train all staff members to ensure data quality. The researchers will record data in case report forms (CRFs) and sign them. CRFs will not be available for direct alteration. The researchers will need to report to the primary investigator if they need to alter a CRF. Any changes to a CRF will need to be signed and dated. Data administrators will enter data into a computer. The CRFs and computers will be locked in the research center, to which only the primary investigator will have a key. The original data will be kept in the research center rather than published. The data will be accessible through the research center upon reasonable request.

The data monitoring and management committee will be composed of the Scientific Research Department of the First Affiliated Hospital of Guangzhou University of Chinese Medicine. This committee will independently review and monitor the research data. The South China Acupuncture Research Center Clinical Subcenter will form a quality monitoring committee. These committees will visit our clinical research center every 6 months to review and monitor the trial. The study group will run a conference to review this trial every month.

### Safety monitoring

To protect the rights and interests of patients, all patients will be informed of the potential benefits and risks of the trial before they enroll. After confirming that the patient understands the relevant content, we will ask the patient to sign an informed consent form. Patients who do not sign the informed consent form will not be admitted to the study.

Acupotomy may trigger adverse events, including dizziness and local hematoma. If dizziness occurs during treatment, the doctor will stop the treatment immediately. Then, the doctor will lay the patient down on the pillow and provide warm water. If hematoma occurs, the patient will be instructed to apply a local cold compress and switch to a hot compress after 24 h to promote dissipation and absorption of the blood. If an adverse drug reaction occurs, the drug will be discontinued. Active clinical observation and symptomatic treatment will be carried out.

The Ethics Committee of the First Affiliated Hospital of Guangzhou University of Chinese Medicine will monitor the safety of this trial and provide advice (e.g., endpoint adjustment) if necessary. When an adverse event occurs in a patient, the researchers will record the details, such as the time, severity, duration, treatment measures, and event outcomes. The responsible doctor will determine the causal relationship between the treatment method and the adverse event and decide whether the patient will be allowed to continue the study. Relevant information will be reported to the ethics committee on the same day.

The following are the criteria for terminating a patient from the study: (1) the patient has experienced a serious adverse event, the patient has requested to suspend participation in the trial, or the trial operator believes that it is necessary for the patient to be terminated from the trial; (2) during the study, the patient is found to have a systemic disease that could not be detected before the start of the clinical trial; (3) for other reasons, the researchers believe that the patient is not suitable for continued treatment; and (4) the patient has privately received other interventions that may affect the outcomes.

### Statistical analysis

Per-protocol subject analysis and intention-to-treat analysis will be used to analyze the efficacy. The safety analysis set will be used to perform the safety evaluation.

We will develop a statistical analysis plan with a statistician. The statistical software package R 3.4.3 will be used to perform a descriptive statistical analysis, exploratory analysis, and dropout analysis. The results will be considered statistically significant when the *P* value is less than 0.05. In the intention-to-treat analysis, when data are missing, the last observation will be used for interpolation. Then, we will perform a sensitivity analysis for the result.

For continuous variables that follow or meet the assumptions of a normal distribution and homogeneity of variance, we will use Student’s *t* test; otherwise, the Mann-Whitney test or Wilcoxon test will be used. A chi-square test will be performed for discrete or categorical variables. When analyzing data obtained by repeated measurements, we will use analysis of variance or analysis of covariance.

For the safety analysis, we will first perform a descriptive analysis. Subsequently, the incidence of adverse reactions will be compared between the two groups using a chi-square test. Causality will be considered in the comparison regarding the severity of adverse reactions and the needle-knife operation. If there are a large number of adverse reactions, the relationship between the intervention time and baseline characteristics will be analyzed. We will use the correlation coefficient to analyze these relationships.

## Discussion

KOA is a chronic disease involving pain, swelling, stiffness, restricted movement, popping, or deformity [29]. It is associated with sex, age, body weight, physical activity, occupation, and biochemical factors [30]. KOA usually occurs in females or elderly individuals. Most KOA patients are overweight or obese. One in every seven cases of KOA is attributable to work, which indicates that occupation is associated with KOA.

There are three views regarding the pathological mechanism of KOA [31]. First, mechanical loading is considered to cause changes in biological stress, which can damage knee cartilage. Furthermore, synovial inflammation is considered to induce matrix metalloproteinase overexpression in cartilage, which can also damage knee cartilage. In addition, some adipokines, such as leptin and adipsin, are known to contribute to the pathological mechanism in terms of triggering cartilage impairment.

The guideline has provided different recommendations for KOA in clinical practice [32]. Physicians always use paracetamol (acetaminophen) and nonsteroidal anti-inflammatory drugs (NSAIDs) (celecoxib, indomethacin, etc.) to address KOA symptoms, according to the guidelines [4, 33–35]. However, patients cannot use these drugs for extended periods. Paracetamol (acetaminophen) does little to improve patients’ pain, stiffness, and reduced physical function [36]. In addition, paracetamol (acetaminophen) causes hepatotoxicity [37] and adverse reactions in the kidney, cardiovascular system, and gastrointestinal tract with long-term use [38]. NSAID use also increases the risks of adverse cardiovascular outcomes [39] and upper gastrointestinal complications [40]. Hence, nonpharmacological therapies play an essential role in treating KOA [41]. There have been some clinical studies of traditional Chinese medicine strategies in treating KOA, such as acupuncture [42], moxibustion [43], tai chi, and massage [44]. Compared with other nonpharmacological therapies, acupotomy is a better choice for the treatment of KOA. It can be applied less frequently, which is good for patient compliance. In addition, acupotomy can not only relieve pain but also improve joint mobility.

As a special type of acutherapy, acupotomy can improve KOA symptoms via different pathways. Acupotomy can restore the biomechanics of the knee by reshaping the physical environment in the knee [45]. Acupotomy can also curb inflammation [46, 47]. The reduction in IL-1β, IL-6, and TNF-α suppresses the expression of MMP-1, MMP-3, and MMP-13, which is conducive to preserving knee cartilage. In short, acupotomy promotes chondrocyte repair and regulates cartilage metabolism [48].

Clinical research has been performed to evaluate the efficacy of acupotomy in treating KOA [9, 10]. However, the design of both of these former trials was inadequate. In both studies, acupuncture was selected as a comparison, which failed to demonstrate the powerful efficacy of acupotomy. One study [11] used inappropriate randomization procedures and did not use blinding, while another [12] had a small sample size and used only a scale as an outcome. Thus, we designed a stricter study to explore the efficacy and safety of acupotomy in treating KOA with sham acupotomy as a comparison. In addition, to the best of our knowledge, this will be the first single-blind study on acupotomy. Moreover, we will also use clinical outcomes (WOMAC, VAS score, joint mobility and quality of life) and surrogate outcomes (musculoskeletal ultrasound findings, X-ray examination findings, plantar pressure distribution testing, maximum knee circumference).

Our exclusion criteria are relatively strict and may exclude many patients in clinical practice. In this manner, we will be able to enroll patients without many confounders, and the results will represent a powerful conclusion regarding the internal validity of this study. However, the external validity of this trial is not strong, which means the data may not be eligible for extrapolation.

This study will have some limitations. First, we will only enroll patients with level I-III KOA; thus, we will not be able to observe the effect of acupotomy on KOA of other levels in patients. Second, we selected a relatively short-term follow-up period for evaluating a chronic arthritis condition, preventing the monitoring of long-term effects. Third, this will be a single-blind study, which means we will not be able to avoid caregivers’ impact. Furthermore, as we will use the last observation carried forward method to interpret findings, if the rate of missing data is high, the stability of the data analysis results after interpolation will become low, potentially even distorting the results of the original data analysis due to bias. Last, this study will be performed in China, so the conclusion may not be suitable for application in patients of other ethnicities.

In summary, we will perform a parallel, block-randomized, single-blind, sham-controlled trial to evaluate the efficacy and safety of acupotomy in KOA patients.

## Trial status

This study will begin in June 2020. The study protocol is version 3.0 (2020.4.27). We will recruit participants from June 1, 2020, to June 30, 2022.

## Supplementary Information

**Additional file 1.** SPIRIT 2013 Checklist: Recommended items to address in a clinical trial protocol and related documents.

**Additional file 2.**

## Data Availability

Not applicable. We have no datasets included in this study protocol. In this research, volunteers will provide informed consent to ensure that data regarding blood indicators can be used in this study, and these data are not applicable to other channels. The results of this study will be communicated with others via a peer-reviewed journal. The full protocol will be accessible upon reasonable request after the trial is complete.

## Abbreviations

KOA: Knee osteoarthritis
WOMAC: Western Ontario and McMaster Universities Osteoarthritis Index
VAS: Visual analog scale
CRF: Case report form
SF-36: 36-Item Short-Form Health Survey
