# Supplementary material for: Effect of acupotomy in knee osteoarthritis patients: study protocol for a randomized controlled trial
Source: Trials. 2021 Apr 20;22:295. doi: 10.1186/s13063-021-05247-z (PMC8056725; doi:10.1186/s13063-021-05247-z)
Supplement: Supplementary file 2 — Additional file 2. [file 13063_2021_5247_MOESM2_ESM.docx]

The specific operation of the needle knife is as follows: the incision line is consistent with longitudinal axis of the lower limbs. The body of needle knife is perpendicular to the skin, and the needle knife is introduced strictly in accordance with the four-step operation procedure of fixed point, orientation, pressure, and penetration. After reaching the corresponding anatomical position, doctor performs the needle knife Technique. The needle knife technique can be Summarized as peeling and cutting. Among them, the peeling techniques include horizontal peeling and fan peeling to further increase the degree of loosening of adhesions, scars and other tissues. The horizontal stripping method means that the needle blade body is centered on the skin, and the needle knife’s edge makes a horizontal arc movement in the body; the fan-shaped stripping method is to penetrate the needle knife into the two layers of adhesion tissue, between the two layers of tissue (there are large areas of adhesive lesions) to strip the tissue in a fan-shaped trajectory. The cutting methods include longitudinal cutting method and lifting cutting method to eliminate local abnormal stress. Longitudinal cutting method is that the needle knife body is centered on the skin, and the needle knife’s edge makes a longitudinal arc movement in the body. Mainly use the edge and the part close to the edge as the active part to cut the lesion tissues such as adhesions; the lifting cutting method is to cut the first notch when the blade reaches the lesion, and then when the needle knife is lifted to the outside of the lesion, and then down Insert into the lesion, cut the second notch, generally 3 cuts are appropriate. During the operation, the needle blade should be parallel to the muscle fibers, nerves, and blood vessels to prevent unnecessary damage. After all the operation points are completed, the surgical wound is compressed to stanch bleeding and disinfection, and finally applied with a band-aid.
